# Current Status and Emerging Trends in Colorectal Cancer Screening and Diagnostics

**DOI:** 10.3390/bios13100926

**Published:** 2023-10-13

**Authors:** Shreya Singh Beniwal, Paula Lamo, Ajeet Kaushik, Dionisio Lorenzo Lorenzo-Villegas, Yuguang Liu, ArunSundar MohanaSundaram

**Affiliations:** 1 Lady Hardinge Medical College, Connaught Place, New Delhi 110001, India; 2 Escuela Superior de Ingeniería y Tecnología, Universidad Internacional de La Rioja, 26006 Logroño, Spain; 3 NanoBioTech Laboratory, Department of Environmental Engineering, Florida Polytechnic University, Lakeland, FL 33805, USA; 4 Faculty of Health Sciences, University Fernando Pessoa-Canarias, 35450 Santa Maria de Guia, Spain; 5 Departments of Physiology and Biomedical Engineering, Immunology and Surgery, Microbiome Program, Center for Individualized Medicine, Mayo Clinic, Rochester, MN 55905, USA; 6 School of Pharmacy, Sathyabama Institute of Science and Technology, Chennai 600 119, India

**Keywords:** colorectal cancer, standard practices, non-invasive, diagnostic, microfluidics

## Abstract

Colorectal cancer (CRC) is a prevalent and potentially fatal disease categorized based on its high incidences and mortality rates, which raised the need for effective diagnostic strategies for the early detection and management of CRC. While there are several conventional cancer diagnostics available, they have certain limitations that hinder their effectiveness. Significant research efforts are currently being dedicated to elucidating novel methodologies that aim at comprehending the intricate molecular mechanism that underlies CRC. Recently, microfluidic diagnostics have emerged as a pivotal solution, offering non-invasive approaches to real-time monitoring of disease progression and treatment response. Microfluidic devices enable the integration of multiple sample preparation steps into a single platform, which speeds up processing and improves sensitivity. Such advancements in diagnostic technologies hold immense promise for revolutionizing the field of CRC diagnosis and enabling efficient detection and monitoring strategies. This article elucidates several of the latest developments in microfluidic technology for CRC diagnostics. In addition to the advancements in microfluidic technology for CRC diagnostics, the integration of artificial intelligence (AI) holds great promise for further enhancing diagnostic capabilities. Advancements in microfluidic systems and AI-driven approaches can revolutionize colorectal cancer diagnostics, offering accurate, efficient, and personalized strategies to improve patient outcomes and transform cancer management.

## 1. Introduction

Colorectal cancer (CRC) represents a significant global health burden, ranking as the second most prevalent cause of cancer-related mortality and the third most widespread malignant neoplasm. CRC primarily affects the colon and rectum, which are integral components of the gastrointestinal tract responsible for waste processing and elimination [1]. Recent epidemiological data from 2020 to 2021 reported a mortality rate of 9.4%, accompanied by a noteworthy increase in incidents among the elderly [2]. The literature review highlights substantial evidence indicating that 90% of CRC cases are diagnosed in individuals aged 50 years and older. Additionally, emerging studies have drawn attention to a rising incidence of CRC in younger cohorts, with an estimated occurrence of 10% in this age bracket [3]. This escalating occurrence of CRC has been attributed to various factors, such as aging and lifestyles such as sedentary habits, suboptimal dietary choices, and tobacco use [4,5].

In oncology diagnostics, there has always been a strong emphasis on detecting cancer at an early stage. This principle applies notably to CRC, as the prompt recognition of signs and symptoms associated with CRC during asymptomatic or pre-cancerous stages can profoundly influence treatment modalities (e.g., personalized care) and overall prognosis (e.g., survival rates and enhanced quality of life) [6]. So far, several standard diagnostic modalities have contributed to the early intervention and improved management of CRC and patient outcomes. These modalities encompass screening tests such as fecal occult blood tests (FOBT), fecal immunochemical tests (FIT), and stool DNA tests, as well as standard diagnoses such as colonoscopy, which directly visualizes a biopsy of abnormal tissue or polyps, or computed tomographic colonography (CTC) as a less invasive alternative [7,8]. Furthermore, blood-based biomarkers, such as carcinoembryonic antigen (CEA) and circulating tumor DNA (ctDNA), have also demonstrated promise as non-invasive screening modalities for CRC. Nevertheless, these conventional methodologies are often limited in sensitivity and specificity, and there is a lack of sufficient validation of stool DNA tests. Other practical concerns include the risks associated with the invasive colonoscopy as well as the ionizing radiation exposure inherent in CTC [9].

In recent years, microfluidic technologies have emerged as a promising tool for disease diagnosis [10,11,12]. These platforms enable controlled handling of small volumes and offer rapid sample-to-answer time with high detection sensitivity and specificity, making them highly attractive for CRC screening and diagnosis. Integrating microfluidics into CRC diagnostics would enable the detection of low-abundance-specific biomarkers or genetic mutations associated with CRC, minimizing the likelihood of false-positive or false-negative results. Moreover, microfluidic platforms can precisely manipulate bacterial and human cells with single-cell resolution, offering the potential to shed new light on the intricate mechanisms underlying CRC development and progression through the analysis of the genome and transcriptome [13,14,15]. As a result, leveraging microfluidic technologies, it is the promise of future novel diagnostic approaches to include early detection, treatment response monitoring, and minimal residual disease detection in CRC patients to better ensure long-term remission. However, to make these new methods broadly available to a wide range of populations, it is imperative to overcome key barriers such as standardization, validation, scalability, and regulatory approval to effectively translate microfluidics into clinical practice and fully exploit its potential in CRC research and patient care.

**Figure 1:**
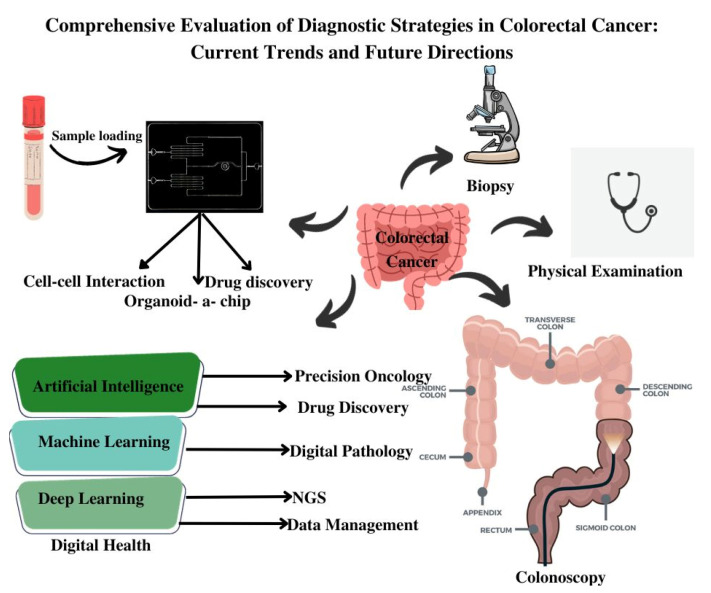
Schematic Illustration of Different Diagnostic Modalities for Colorectal Cancer.

This review provides a comprehensive comparison of the standard practices in CRC diagnosis and treatment. It offers a critical assessment of the strengths and limitations of the established approaches, highlighting the need for more innovative and effective strategies, as illustrated in Figure 1. In this context, the article delves into the emerging field of microfluidics, which has garnered significant attention as a promising avenue for advancing research and diagnosis in CRC. Lastly, the article sheds light on the integration of artificial intelligence (AI) in the field of CRC, highlighting the advancements, challenges, and future directions in this exciting area.

## 2. Emerging Paradigms in CRC Diagnostics

**Figure 2:**
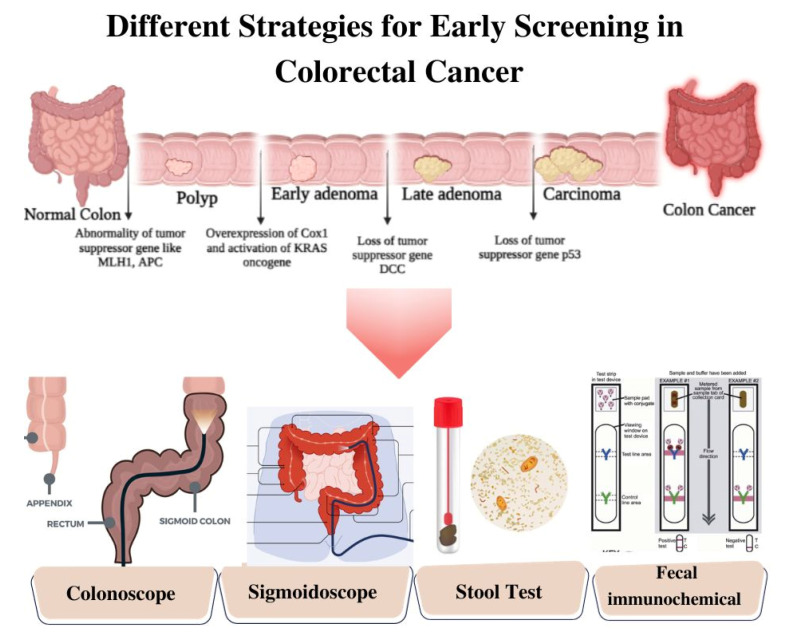
Schematic Illustration of Different Strategies of Screening for Colorectal Cancer.

CRC diagnostic methods are typically classified into invasive and non-invasive modalities (Figure 2). Invasive tests encompass colonoscopy, flexible sigmoidoscopy, virtual colonoscopy (CT colonography), and double-contrast barium enema [16]. These procedures involve direct access to the colon and rectum to visually examine the colorectal region, identify pathological features, and perform biopsies when required. Conversely, non-invasive tests employ approaches such as fecal occult blood test (FOBT), fecal immunochemical test (FIT), and stool DNA test (MT-sDNA) [17]. These non-invasive techniques analyze stool samples to detect the presence of blood, genetic markers, or DNA alterations associated with CRC. The selection of invasive or non-invasive methods is contingent upon individual risk factors, symptoms, and the recommendations of healthcare providers. Both invasive and non-invasive strategies play pivotal roles in the diagnosis and management of CRC, leading to improved outcomes and tailored treatment modalities. The below discussion gives a brief insight into different invasive and non-invasive methods.

## 3. Unleashing the Potential of Stool-Based Diagnostics

Stool tests have emerged as a valuable screening modality for CRC detection. These tests aim to identify specific biomarkers, such as blood or DNA alterations in the stool of the asymptomatic patients. One commonly used stool test is the fecal occult blood test [18]. FOBT can be guaiac-based (gFOBT) or immunochemical (FIT). The gFOBT utilizes a guaiac reagent to detect the presence of blood in the stool through a chemical reaction that is characterized as a peroxidase-like reaction to discern the existence of blood within stool specimens. In this biochemical process, the guaiac reagent engages with the heme moiety contained within hemoglobin, an essential constituent of erythrocytes (red blood cells). This interaction precipitates a chromogenic alteration, often manifesting as a blue coloration, which serves as an indicator for the presence of occult (concealed) blood in the fecal matter, while FIT employs antibodies to specifically detect human hemoglobin. Among these, FIT demonstrates up to 79% sensitivity and 94% specificity, reflecting its efficacy as a robust screening modality for detecting CRC prior to symptoms [19] and contributing to a 22% reduction in CRC incidences [20].

In addition to FOBT, the stool DNA test, also referred to as a “multi-targeted stool DNA” test, has been investigated as another promising stool-based screening method for CRC. The test focuses on detecting specific DNA markers associated with CRC in stool samples. By analyzing alterations in genes or DNA regions known to be linked to CRC, these tests can identify specific molecular changes that are indicative of CRC or advanced adenomas. These genetic alterations include mutations, methylation patterns, or other biomarkers associated with CRC development [21]. The stool DNA test offers a sensitivity of 92% and a specificity of 87%, outperforming FIT detection [22].

Various studies have used stool-based diagnostic approaches. For instance, based on the data from a comprehensive colon cancer screening program, Vakil et al. [23] retrospectively evaluated the effectiveness of multi-target stool DNA testing for colon cancer prevention in a comprehensive healthcare system. The primary outcomes evaluated were the detection rate of colon cancer and advanced precancerous lesions, as well as the rate of interval cancers and follow-up colonoscopies. The findings supported the clinical utility and effectiveness of multi-target stool DNA testing for colon cancer prevention. From the result, it was demonstrated that the MT-sDNA test for colorectal cancer screening had limitations in terms of its detection rate, a high false positive rate, and challenges related to patient adherence to follow-up colonoscopy. These findings suggest that it may not be as effective as high-quality colonoscopy for detecting CRC and advanced adenomas, and these factors need to be considered when adopting this screening strategy [23]. Similarly, Dolatkhah et al. [24] demonstrate a systematic review and meta-analysis to assess the diagnostic accuracy and reliability of multi-target stool DNA testing as a non-invasive method for CRC screening. The findings of the Mt-sDNA test revealed that it possesses moderately good diagnostic accuracy for the detection of CRC and advanced adenoma (AA). Specifically, the test showed a sensitivity of 89% for CRC, 51% for AA, and 76% when detecting a combination of CRC and AA. In terms of specificity, it demonstrated values of 91% for CRC, 89% for AA, and 90% for the combined detection of CRC and AA. However, when compared to colonoscopy, which is considered the gold standard for colorectal cancer screening, the Mt-sDNA test still falls short in terms of both sensitivity and specificity. While it offers a non-invasive alternative for diagnosis, its diagnostic performance is not as robust as colonoscopy, which has higher sensitivity and specificity rates [24].

Despite the numerous advantages of stool tests, this diagnostic modality demonstrates a sensitivity of only 42% in detecting advanced polyps [25]. Moreover, these tests present challenges in sample collection, affecting their practicality and widespread adoption and limiting their effectiveness in identifying such precancerous growths. Consequently, for comprehensive evaluation and confirmation of diagnoses, visualizing the colon and rectum, removing polyps or cancerous growths, and further assessing through colonoscopy remain imperative steps in CRC screening to ensure accurate and reliable results.

## 4. Colonoscopy: A Comprehensive and Minimally Invasive Procedure for Colorectal Examination and Diagnosis

Colonoscopy is a vital procedure in CRC diagnosis and surveillance. As the gold standard screening and diagnostic modality, this technique enables the direct visualization and thorough examination of the entire colon and rectum, facilitating the identification of early-stage CRC lesions [26]. Through a flexible colonoscope equipped with an advanced imaging system, healthcare professionals visually inspect the mucosal lining of the colon and rectum, enabling the detection of aberrant tissue, polyps, or tumors that may be indicative of CRC. Furthermore, colonoscopy facilitates the procurement of tissue samples (biopsies) for histopathological assessment, aiding in accurate staging and determining the aggressiveness of the disease. In addition to diagnostics, colonoscopy also assumes a critical role in post-treatment surveillance, enabling the detection of potential recurrences or the development of new polyps in individuals with a history of CRC. The overall effectiveness of colonoscopy can be reflected by its sensitivity range of 93–94% and specificity up to 99.8%, making it an indispensable cornerstone in CRC prevention, early detection, and ongoing monitoring [24].

However, colonoscopy has several challenges and drawbacks, such as the risk of contrast allergies and perforation. The procedure also demands bowel preparation, causing extra burdens for patients (especially the old and the vulnerable) and incurring high utilization of medical resources and thus expenses. While colonoscopy can detect numerous precancerous polyps, only a minority of these lesions possess the potential to progress into cancer. The lack of evidence on the specific polyps posing an increased risk results in shorter surveillance intervals for individuals with polyps, leading to escalated demand and strain on the healthcare system without clearly defined benefits. Moreover, financial and psychosocial barriers may adversely affect patient adherence to colonoscopy, making it less favorable compared to other screening methods such as FIT. Consequently, in programmatic screening endeavors, colonoscopy is best used as the second step in a two-stage screening cascade [27].

## 5. Advancing Colorectal Cancer Screening: Sigmoidoscopy and CT Colonography as Powerful Diagnostic Techniques

Sigmoidoscopy and CT colonography have emerged as valuable diagnostic modalities for CRC evaluation. Sigmoidoscopy, a minimally invasive procedure, allows for the visualization and examination of the rectum and sigmoid colon. Using a flexible sigmoidoscope, the presence of abnormal tissue, polyps, or tumors in the lower portion of the colon can be assessed. The technique serves as an effective screening tool for detecting CRC and its precursors, particularly in the distal colon [28]. On the other hand, CT colonography, also known as virtual colonoscopy, employs computed tomography imaging to generate high-resolution images of the entire colon and rectum. These images are reconstructed using specialized software, enabling the identification and characterization of polyps and lesions. As CT colonography is less invasive than conventional colonoscopy and does not require sedation, it demonstrates its potential as a screening modality for CRC, particularly in situations where conventional colonoscopy may be contraindicated or poorly tolerated [29].

CT colonography demonstrated promise in detecting adenomas with a size of ≥6 mm, showing a sensitivity of 76% and a specificity between 89% and 91%. Additionally, for larger adenomas (≥10 mm), CT colonography demonstrates a pooled sensitivity of 67% to 94% and a specificity ranging from 96% to 98%. These findings suggest that CT colonography has the potential to effectively identify larger adenomas, which are of significant clinical importance. However, CT colonography has limitations in detecting sessile and flat polyps, which may lead to missed detections in certain cases [30,31,32].

Table 1 below gives a comprehensive summary of the conventional screening modalities used for CRC detection, offering insights into their individual strengths and limitations.

## 6. Revolutionizing Colorectal Cancer Diagnosis: Expanding Horizons with Biomarker-Based Detection

Although conventional screening modalities played a crucial role in CRC detection, their limitations spurred significant interest and extensive research in exploring biomarker-based detection strategies. A recent study reported [37] a comparative analysis of a colonoscopy, a fecal immunochemical test, and a risk-adapted approach in a colorectal cancer screening trial (TARGET-C) to compare three distinct methods of CRC screening. The study randomized the participants into three groups, with each group receiving the screening method mentioned above. Subsequently, the authors evaluated the effectiveness, accuracy, and overall outcomes of each approach in detecting CRC or precancerous lesions. The findings indicated that the risk-adapted approach demonstrates feasibility and cost-effectiveness as a population-based strategy for CRC screening and thus holds promise as a valuable complement to the well-established one-time colonoscopy and annually repeated FIT screening.

However, these methods are not without challenges. For example, invasiveness may lead to patients’ discomfort and health concerns, including mild skin complications and gastrointestinal disturbances after diagnostic procedures such as radiation therapy. Moreover, several factors, including limited awareness, reluctance to undergo invasive interventions, financial constraints, and diverse geographic or socio-economic barriers, contribute to the limitations of conventional CRC screening approaches. As a result, there is a growing demand for novel and patient-friendly approaches that are non-invasive or minimally invasive, aimed at improving patient acceptance and adherence to recommended screening protocols [38].

Biomarker-based detection offers promising solutions to meet these demands. By targeting specific molecular or genetic alterations associated with CRC, these tests can provide a less invasive and more patient-friendly screening. For instance, blood-based tests involve simple blood draws, obviating the need for uncomfortable bowel preparation or instrument insertion. Similarly, stool-based tests can be conveniently conducted at home, mitigating the challenges associated with conventional screening [39].

**Figure 3:**
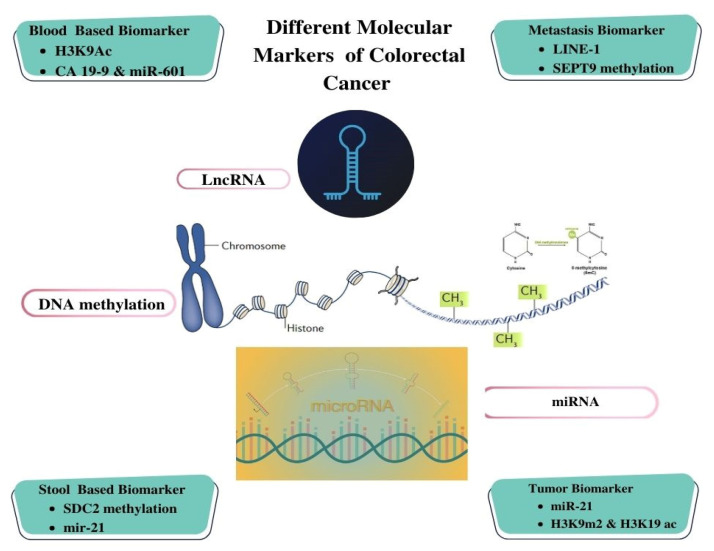
Illustration of Different Molecular Biomarker-based Detection for Colorectal Cancer.

Diverse types of biomarkers of CRC have been investigated, including circulating tumor DNA (ctDNA), microRNAs, DNA methylation markers, and protein-based markers. ctDNA, fragments of tumor-derived DNA present in the bloodstream, enables the detection of specific genetic mutations or alterations associated with CRC, as represented in Figure 3 [40,41,42]. MicroRNAs, small non-coding RNA molecules, exhibit dysregulated expression patterns in CRC and hold potential as informative biomarkers. DNA methylation markers (vimentin, septin 9, p16, APC, mutL homolog 1, and death-associated protein kinase 1), involving epigenetic modifications of DNA molecules, can provide insights into the epigenetic changes implicated in CRC development and progression.

Protein-based markers, such as carcinoembryonic antigen (CEA), have been extensively employed for CRC screening and therapeutic response monitoring [43]. However, CEA is not routinely used as a diagnostic tool for CRC in global clinical practice due to its potential elevation in various inflammatory conditions, such as diverticulitis and inflammatory bowel disease. Moreover, CEA exhibits limited sensitivity but notable specificity as a diagnostic marker.

For instance, Nicholson et al. [44] conducted a meta-analysis that involved data from 52 studies, encompassing a total of 9717 patients. The evaluation of the diagnostic performance of carcinoembryonic antigen (CEA) was carried out across a spectrum of threshold values, ranging from 2 to 40 µg/L. This analysis brought to light the considerable variability in CEA’s diagnostic efficacy contingent upon the chosen threshold. Specifically, at a threshold of 2.5 µg/L, CEA demonstrated a sensitivity of 82% and a specificity of 80%. Conversely, the threshold most frequently utilized, set at 5 µg/L, yielded a sensitivity of 71%, accompanied by an elevated specificity of 88%. Raising the threshold to 10 µg/L resulted in a reduced sensitivity of 68%, but it conferred a notably elevated specificity of 97%. However, the study identified one limitation of CEA-based diagnostics: the absence of a direct correlation between the degree of CEA upregulation and disease prognosis or metastatic progression. Nevertheless, CEA testing remains valuable for monitoring treatment response following chemotherapy or for screening disease recurrence after completing the treatment regimen [44].

Besides, exosomes have emerged as pivotal entities in the landscape of CRC. These diminutive vesicles, originating from CRC cells and various components within the tumor microenvironment, serve in multifaceted roles concerning CRC’s progression, diagnostic prospects, and therapeutic avenues. Notably, their ability to convey distinct genetic material and disease-relevant proteins renders CRC-derived exosomes of considerable scientific significance. These exosomes, detectable in bodily fluids such as blood and stool, present an opportunity for non-invasive and easily accessible diagnostic information. Researchers are actively engaged in delineating distinctive microRNA profiles and protein markers encapsulated within these exosomes, thereby facilitating the development of highly sensitive and specific diagnostic assays [45,46]. The comprehensive analysis of the molecular content housed within CRC-derived exosomes holds the potential to enable the early-stage detection of CRC, facilitate disease progression monitoring, and allow for the customization of treatment strategies. This promising endeavor signifies a significant advancement in the realm of CRC diagnosis and patient care within the scientific domain.

The aforementioned biomarkers emerged as a promising avenue for enhancing screening, diagnosis, and treatment monitoring and can offer valuable insights into the underlying genetic and epigenetic alterations implicated in CRC. The advent of high-throughput technologies, such as next-generation sequencing and advanced proteomics, is revolutionizing biomarker discovery and validation, enabling the identification of novel biomarkers with enhanced sensitivity and specificity. Furthermore, the integration of biomarkers into non-invasive diagnostic approaches, such as blood-based tests and stool-based assays, has expanded their clinical utility and patient acceptance. Through the integration of advancements in molecular biology and diagnostic technologies, ongoing research endeavors aim to identify novel markers and optimize their clinical applicability [47,48]. The incorporation of these biomarkers into routine clinical practice has the potential to revolutionize CRC management, fostering improved patient outcomes, refined treatment decisions, and a more targeted approach to combating this complex disease [49].

Biomarker-based approaches for CRC detection present substantial potential but are encumbered by formidable impediments, encompassing the requisite for robust validation, the intricacies associated with CRC’s intrinsic heterogeneity, the imperative to ensure cost-effective accessibility, the imperative to mitigate the incidence of both false positives and negatives, the necessity for standardization of biomarker assays, ethical considerations intrinsic to the gathering and analysis of sensitive genetic data, the exigency for seamless integration into existing screening paradigms, and the essentiality of efficacious follow-up and treatment modalities. Despite these exigencies, ongoing scientific inquiry, technological advancements, and collaborative endeavors portend the prospect of a substantial metamorphosis in CRC screening, diagnostic, and therapeutic paradigms, underpinned by the assimilation of biomarkers as an integral component of customary clinical practice.

## 7. Nanotechnology-Driven Innovations in CRC Diagnosis: Unveiling the Power of Nano-Enabled Tools

Conventional methods demonstrated good sensitivity but could miss some early-stage cases or precancerous lesions. Therefore, the discriminatory power to distinguish individuals with and without CRC is not robust until the onset of primary symptoms, which can delay diagnosis and treatment [50]. It is therefore imperative to increase the sensitivity and specificity of CRC screening methods to detect CRC at precancerous stages, prompting timely and successful interventions [51,52]. Meanwhile, high specificity is equally crucial to minimize false-positive results, as these can lead to unnecessary follow-up procedures that not only increase healthcare costs but also cause undue anxiety and stress for patients. Ensuring high specificity in CRC screening can better ensure that medical resources are allocated to only patients with positive results [9].

**Figure 4:**
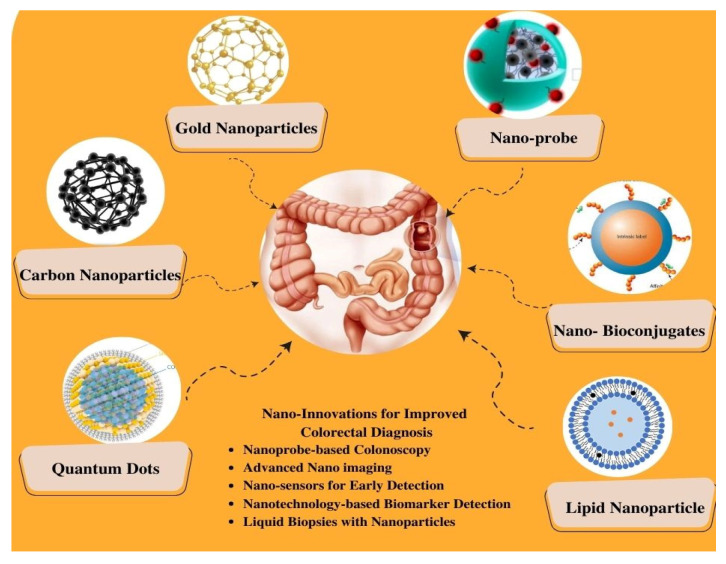
Schematic Representation of Nano-Enhanced Imaging Techniques for Colorectal Cancer Diagnosis.

In recent years, nanotechnology has emerged as a rapidly evolving field in the advancement of novel diagnostics for CRC. The unique properties and capabilities of nanomaterials offer new avenues for the sensitive and specific detection of CRC biomarkers, enabling early diagnosis and personalized treatment strategies, as represented in Figure 4 [53]. The integration of nanotechnology in CRC diagnostics offers unparalleled precision and efficacy, surpassing conventional techniques. This is attributed to the unique physicochemical properties inherent in nanomaterials, which enable efficient biomarker capture and amplification due to their substantial surface area-to-volume ratio [54,55]. Moreover, nanotechnology-based strategies can enable multiplexed analysis in a single assay. Furthermore, these technologies can be customized to accommodate diverse sample types, including blood, tissue, and stool, extending their applicability in CRC diagnostics [56,57]. Here, we focus on nanoparticles, such as quantum dots, gold nanoparticles, and magnetic nanoparticles; dendrimers have been extensively explored for their potential in CRC diagnostics.

### 7.1. Quantum Dots (QDs)

Quantum dots (QDs) are nano-scale fluorescent semiconductor crystals renowned for their unique optical characteristics, especially their exceptional brightness, photo stability, and tunable emission spectra, enabling multiplexed imaging capabilities [58]. QDs can be conjugated with ligands, facilitating their binding to target molecules such as cancer cells or tumor vasculature and thus enabling precise and specific imaging of specific molecular targets [59]. Therefore, QDs present a compelling opportunity for the advancement of sophisticated imaging modalities in CRC research and diagnostics [55].

For instance, Gazouli et al. [60] developed a QD-labeled magnetic immunoassay (QD-MIA) technology that combines QDs and magnetic separation strategies to detect circulating CRC cells. Briefly, this platform introduced a novel, cost-effective methodology for the identification of circulating CTC cells in human biological specimens. This approach involved the utilization of magnetic bead isolation followed by QD fluorescence-based detection, which demonstrated a verified minimum detection limit of 10 DLD-1 CRC cells/mL as quantified through spectrofluorometry. Furthermore, the robustness and reliability of this method were comprehensively assessed through the integration of fluorescence-activated cell sorting analysis and real-time RT-PCR. The findings of the studies underscored the establishment of a straightforward, exceptionally sensitive, and efficient technique with substantial potential for the identification of CTCs in patients with CRC. Notably, the adaptable nature of this method for targeting a wide array of protein markers, whether associated with CTCs or the host organism, implied its versatility and suitability for a diverse range of applications that extended beyond the confines of colorectal cancer detection [60]. Likewise, Carbary-Ganz et al. [61] developed a highly specific and sensitive imaging tool for CRC by exploiting the overexpression of VEGFR2 in the CRC vasculature. In this work, the synthesized QDs were conjugated with VEGFR2-targeting ligands, allowing for the selective binding of the QDs to VEGFR2 in CRC tissues. The study found that the QD655-VEGFR2 agent demonstrated a sensitivity of 85.7% and a specificity of 91.3% in detecting VEGFR2 expression in colorectal cancer. On the other hand, the negative control contrast agent, QD655-IC, had a sensitivity of 5.6% but showed 100% specificity. This suggests the VEGFR2-targeted QDs demonstrated excellent imaging capabilities, providing enhanced contrast and improved visualization of CRC lesions compared to non-targeted QDs or conventional imaging agents [61].

### 7.2. Carbon-Based Nanoparticles

Carbon-based nanoparticles include carbon nanotubes, graphene, and carbon dots and offer several advantages for CRC diagnosis. Compared with QDs and gold nanoparticles, the biocompatibility of carbon nanoparticles is a notable characteristic that ensures their safe administration in vivo without significant adverse health effects [62]. In addition, these nanoparticles exhibit a large surface area and high electrical conductivity, which makes them ideal for functionalization with specific targeting ligands or biomolecules [63], and they can be integrated with electrical-based sensors such as impedance and electrochemical sensors that are common in point-of-care platforms. For example, Yan et al. [64] assessed the efficacy of carbon nanoparticles in the detection of lymph node metastasis in early-stage (T1–2) CRC. The patient cohort comprised individuals who underwent surgical resection for T1–2 CRC, with carbon nanoparticles administered via submucosal injection around the tumor site. The results revealed that these nanoparticles effectively traced lymphatic drainage and accurately identified sentinel lymph nodes in T1–2 CRC, employing near-infrared fluorescence imaging. Notably, the carbon nanoparticle-guided sentinel lymph node biopsy exhibited a superior detection rate for lymph node metastasis compared to conventional histopathological examination, demonstrating heightened sensitivity of 91.67% and a specificity value of 100% [64]. Similarly, for another group, Zhang et al. [65] demonstrated the efficacy of carbon nanoparticles in identifying sentinel lymph nodes and evaluating lymph node metastasis in elderly patients with CRC who underwent surgical procedures using the same approach. The results showed the successful guidance of carbon nanoparticles in identifying sentinel lymph nodes, enabling accurate evaluation of lymph node metastasis in elderly patients with CRC. These findings indicate the promise of carbon nanoparticles in enhancing lymph node staging precision, aiding treatment decision-making, and reducing the extent of lymphadenectomy in CRC patients [65].

In another example, Cai et al. [66] studied the efficacy of activated carbon nanoparticle suspension and methylene blue for lymph node staining in CRC. In the in vivo experiment, activated carbon nanoparticle suspension was administered through lymphatic vessel injection in mice with CRC, and the successful staining of the regional lymph nodes allowed for their visualization. Additionally, in the in vitro experiment, methylene blue was employed to stain lymph nodes obtained from CRC patients. The comparable staining efficacy between activated carbon nanoparticle suspension and methylene blue indicated their effectiveness in highlighting the lymph nodes, suggesting that activated carbon nanoparticles are a promising alternative to methylene blue for lymph node staining in CRC [66]. In another study by Wang et al. [67] prior to surgery, carbon nanoparticles were injected into the submucosal layer surrounding the tumor site during endoscopic examination. Subsequently, laparoscopic surgery was performed, and the carbon nanoparticles served as a guide for the identification and tracking of lymph nodes. The results demonstrated successful preoperative endoscopic localization of CRC utilizing carbon nanoparticles in all enrolled patients during surgery. Overall, the integration of carbon nanoparticles into laparoscopic procedures provided real-time visualization and improved the precision of lymph node dissection. This innovative approach holds significant potential for optimizing surgical outcomes, reducing complications, and enhancing the overall management of patients with CRC [67].

### 7.3. Lipid-Based Nanoparticles

Lipid-based nanoparticles, which comprise lipid materials such as phospholipids or cholesterol, offer several advantages for CRC diagnosis [68,69]. As with many other nanoparticles, lipid-based nanoparticles can be functionalized with specific targeting ligands or antibodies that selectively recognize CRC biomarkers or tumor-associated antigens [70]. Moreover, lipid-based nanoparticles can encapsulate imaging agents, such as fluorescent dyes or contrast agents, facilitating enhanced visualization of CRC lesions using various imaging techniques, including fluorescence imaging or MRI. The unique attributes of lipid-based nanoparticles, encompassing their biocompatibility, stability, and capacity to carry imaging payloads, render them well-suited for the precise and non-invasive diagnosis of CRC [71]. For instance, Alrumaihi et al. [72] aimed to enhance the bioavailability of diallyl trisulfide (DATS), a bioactive compound with known anticancer properties, by encapsulating it in polyethylene glycol-coated liposomes (DATSL) and combining it with doxorubicin (DOXO)-encapsulated liposomes (DOXL). DATSL and DOXL exhibited significant sensitivity in inhibiting colon cancer cell proliferation, both individually and in combination, with a synergistic effect observed at specific concentration combinations, lowering the IC50 doses of DATS and DOXO by over 8- and 14-fold, respectively. In an AOM-induced colon cancer mouse model, high-dose DATSL pretreatment followed by DOXL chemotherapy effectively inhibited cancer promotion, with DATSL and DOXL exhibiting approximately 93% and 46% entrapment efficiency, respectively. Additionally, molecular docking analysis identified potential interactions with cancer-related proteins, such as MMP-9, suggesting a mechanism for DATS action. This study presents a promising approach for colorectal cancer prevention and treatment, highlighting the potential of DATSL and DOXL as a novel and efficient therapeutic strategy [72].

Likewise, Xia et al. [73] demonstrated a regorafenib (REG)-loaded self-assembled lipid-based nanocarrier (SALN) to address the challenges associated with the limited oral bioavailability and rapid systemic clearance of anticancer drugs. SALN, formulated with lipid-based excipients, leveraged the natural lipid transport mechanisms within enterocytes to enhance the lymphatic absorption of REG in the gastrointestinal tract. The resulting SALN particles exhibited a small average size of 106 ± 10 nm. Mechanistically, SALNs were internalized by intestinal epithelial cells via clathrin-mediated endocytosis and subsequently transported across the epithelium through the chylomicron secretion pathway, leading to a notable 3.76-fold increase in drug epithelial permeability (Papp) compared to the solid dispersion (SD). It was observed that the oral bioavailability of REG from SALN was substantially enhanced, showing a 65.9-fold and 1.70-fold increase compared to coarse powder suspension and SD, respectively, and this enhancement was predominantly attributed to lymphatic absorption. Additionally, SALN extended the drug’s elimination half-life (9.34 ± 2.51 h) compared to SD (3.51 ± 0.46 h), exhibited improved biodistribution in tumor and gastrointestinal tissues, reduced distribution in the liver, and demonstrated superior therapeutic efficacy in a colorectal tumor-bearing mouse model compared to solid dispersion. These findings collectively highlight the potential of SALN as a promising strategy for the treatment of CRC through enhanced lymphatic transport, offering prospects for clinical translation [73].

Nanotechnology-driven advancements in the diagnosis of CRC come with noteworthy limitations that require careful scientific consideration. Foremost among these constraints is the potential for biocompatibility and toxicity quandaries. Nanoparticles employed in diagnostic applications may engender unforeseen interactions within biological systems, culminating in plausible deleterious repercussions. The imperative of ensuring the safety profile of these nanoparticles for in vivo implementation necessitates comprehensive toxicity assessments and rigorous empirical evaluation. Concomitantly, the scaling-up and mass production of nanoscale diagnostic modalities can be encumbered by formidable cost considerations and intricate technical challenges, potentially hindering their ubiquitous integration into clinical contexts. Furthermore, the long-term stability and durability of nanoscale diagnostic platforms mandate meticulous scrutiny to affirm their sustained reliability. The intricate regulatory approval processes governing the utilization of nanotechnology-based diagnostic tools impose substantial temporal impediments, possibly impeding their expeditious assimilation into customary clinical practice. While nanotechnology offers enticing prospects for advancing CRC diagnosis, these intricacies must be judiciously addressed to unlock its full potential in augmenting early disease detection and clinical management paradigms.

## 8. Integration of Microfluidics in CRC: Promising Tools for Precise Diagnosis

Microfluidic technologies can precisely manipulate and control fluids at the microscale and provide a foundation for developing lab-on-a-chip devices. These miniaturized systems offer numerous advantages, including reduced sample and reagent consumption, accelerated reaction times, enhanced sensitivity, and portability [74]. The domain of CRC diagnosis has witnessed noteworthy progressions, notably in the realm of microfluidics as a promising and rapidly evolving field. This section aims to elucidate the manifold applications and advantages of microfluidics in CRC diagnostics [75].

The integration of biosensing into microfluidic platforms for the detection of CRC is a notable endeavor. Electrochemical and colorimetric biosensing modalities are particularly pertinent in this context. In the field of CRC, microfluidics has catalyzed profound transformation by significantly innovating enhance detection and diagnosis modalities. The advent of microfluidics has enabled the isolation and analysis of circulating tumor cells (CTCs) and cell-free DNA (cfDNA) from blood specimens, providing a non-invasive avenue for monitoring cancer progression and treatment response [76,77] detection of genetic mutations, tumor markers, and other biomarkers associated with colorectal cancer, thereby enabling early-stage diagnosis and point-of-care testing. The introduction of microfluidic-based liquid biopsies has had a transformative impact on early cancer detection and personalized medicine strategies, as evidenced by a comprehensive analysis of the existing literature [78,79]. As a result, the utilization of miniaturized microfluidic chips has empowered rapid and highly sensitive detection methods, enabling timely interventions and ultimately leading to improved patient outcomes.

Different detection methods used in the study of CRC encompass the identification of CTCs, isolation and examination of tumor exosomes, analysis of DNA biomarkers, and evaluation of microRNAs in microfluidic devices or lab-on-chip systems. These methodologies provide valuable information about the molecular and cellular characteristics of colorectal cancer, enabling early detection and prognosis determination approaches.

### 8.1. Circulating Tumor Cells Detection

Microfluidic-based methods have gained significant attention in CRC research for the isolation, detection, and analysis of CTCs in patients’ blood samples. By employing immunoaffinity capture, microfluidic platforms selectively capture CT\Cs using specific antibodies immobilized on the device or micro- and nanoscale particles while washing away other blood cells. Various enrichment strategies, such as positive and negative selection, as well as size-based enrichment, are incorporated to enhance CTC enrichment efficiency. Once cells are captured, they can also be subjected to immunofluorescence staining, nucleic acid amplification, and molecular profiling directly on the microfluidic chip, allowing for comprehensive characterization of CTCs.

**Figure 5:**
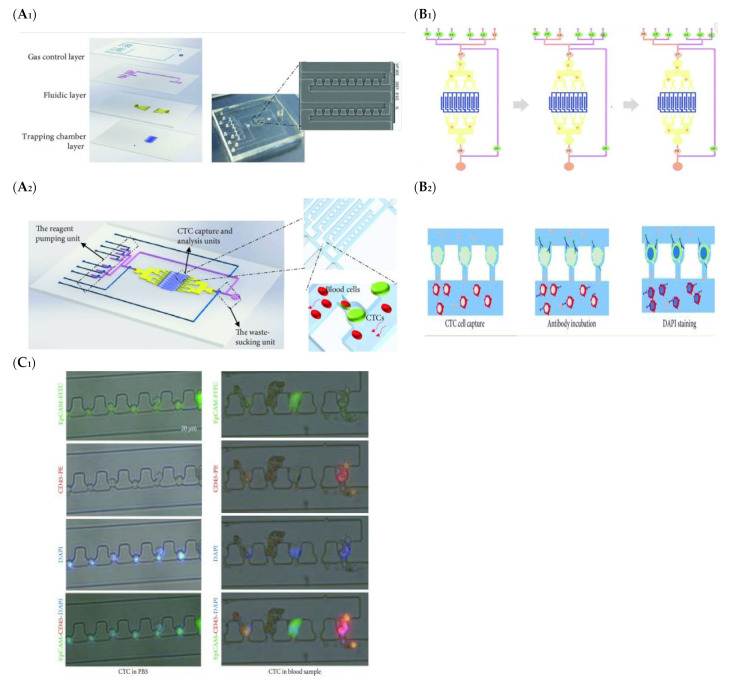
Schematic illustration (**A_1_**,**A_2_**) Integrated microfluidic device for CTC enrichment and analysis (**B_1_**,**B_2_**) Development of Integrated Microfluidic Device for Simultaneous Enrichment and Analysis of Circulating Tumor Cells, (**C_1_**) Comparative Immuno-staining Analysis of Captured Cells in PBS and Human Blood Samples [81]. (Reproduced with permission from Su, W.; Yu, H.; Jiang, L.; Chen, W.; Li, H.; Qin, J, Disease Markers; published by Hindawi, 2019).

Microfluidic-based CTC detection offers a number of advantages, such as increased sensitivity and specificity, reduced sample volume requirements, real-time analysis capabilities, and high-throughput screening potential. Various studies have reported the application of microfluidic-based CTC detection in CRC research. For instance, a microfluidic platform can integrate size-based filtration to isolate CTCs from peripheral blood samples from CRC patients, taking advantage of the size differences between CTCs and blood cells [80]. The captured CTCs could then be counted, analyzed, and characterized, providing valuable information for disease monitoring and treatment response assessment. The platform offers potential as a non-invasive method for CTC detection in CRC patients. Likewise, a similar platform has been used by other groups for the enrichment and detection of rare CTCs in the blood stream of cancer patients, as shown in Figure 5 [81], and the abundance of CTCs provided valuable information about cancer progression and treatment response. However, isolating and analyzing CTCs is still challenging due to their low abundance and heterogeneous nature. To enhance the capture efficiency, a combination of physical and immunomagnetic separation methods can be integrated into the device to enrich CTCs from blood samples. The captured CTCs were then characterized using immunofluorescence staining, allowing for their identification and further analysis. To enhance the accessibility of the platform to minimally trained hands, others designed a microfluidic platform that incorporated an antibody-coated surface for specific CTC capture and integrated automation for enhanced efficiency and reproducibility [82]. The device demonstrated excellent sensitivity and selectivity, enabling the detection and characterization of CTCs from clinical blood samples with high accuracy.

### 8.2. Microfluidic-Based Isolation and Characterization of Tumor Exosomes

In recent years, substantial advancements have been made in the development of exosome detection chips based on microfluidic platforms. These innovative chips offer rapid, sensitive, and high-throughput detection of exosomes, thereby presenting new avenues for diagnostics, biomarker discovery, and therapeutic applications [83]. Exosome detection chips commonly utilize diverse functional components and techniques to optimize the efficiency and specificity of exosome capture and analysis [84]. A prevalent strategy that is involved in surface functionalization of the microfluidic chip includes antibodies or aptamers that exhibit selective affinity towards exosome surface markers, thereby facilitating their targeted capture and separation from other extracellular vesicles or contaminants. Furthermore, these chips facilitate the integration of downstream analytical techniques into the same device. For instance, the captured exosomes can be subjected to further analysis employing fluorescence microscopy, immunoassays, or nucleic acid amplification techniques. Such integrated platforms offered a streamlined workflow, minimizing sample loss and reducing the overall time required for analysis. These chips hold promise in various applications, such as early cancer detection, monitoring disease progression, and assessing treatment response [85].

A number of studies utilized the use of exosomes for CRC, for instance (Li et al., 2023) demonstrate the construction of a novel exosome detection platform using a three-dimensional (3D) porous microfluidic chip. The platform enabled early diagnosis of CRC by detecting SORL1 protein in exosomes. The 3D porous microfluidic chip offered a significant increase in functionalized surface areas to selectively capture exosomes expressing SORL1, a biomarker associated with CRC. Clinical samples from CRC patients and healthy controls were analyzed, and the captured exosomes were subjected to immunoassays and nucleic acid amplification to detect SORL1. The platform exhibited efficacy of up to 90%, accurately distinguishing CRC patients from healthy individuals based on exosomal SORL1 levels. To enable more streamlined sample processing, others presented an advanced lab-on-a-chip platform that combines pre-concentration and detection of CRC exosomes in a single device [86]. The platform utilizes an anti-CD63 aptamer as a recognition element for specific exosome capture. By integrating microfluidic techniques for efficient pre-concentration and incorporating the aptamer for selective exosome binding, the platform demonstrates detection limit of 1457 particles/mL, thus offering a promising approach for the sensitive and streamlined analysis of colorectal cancer exosomes, enabling improved early detection, prognosis, and personalized treatment strategies.

**Figure 6:**
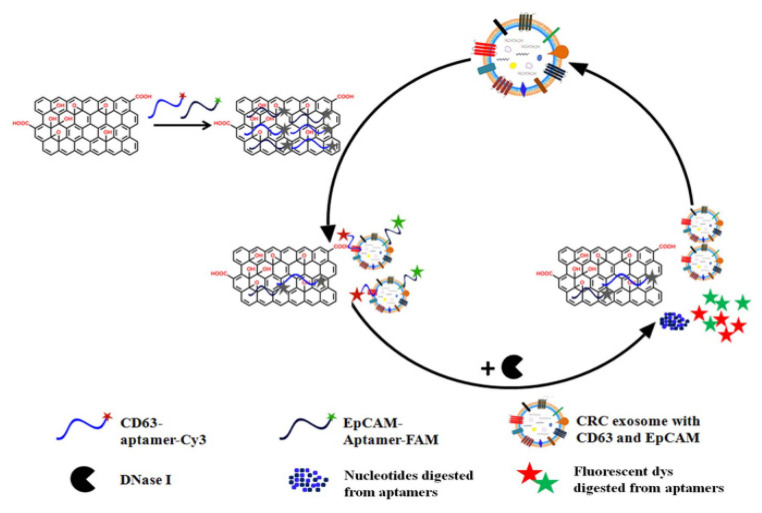
Schematic representation illustrating the underlying mechanism of enzyme-aided fluorescence amplification based on GO-DNA aptamer interactions for exosome detection (Image taken from: Reproduced with permission from Wang, H.; Chen, H.; Huang, Z.; Li, T.; Deng, A.; Kong, J. Ta-lanta; published by Elsevier, 2018 [87].

To further increase the sensitivity of exosome detection, a novel method combines fluorescence signal amplification aided by the DNase I enzyme and graphene oxide-DNA aptamer interactions to capture CRC exosomes [87]. Functionalizing graphene oxide with DNA aptamers targets exosome surface markers with a high sensitivity of 2.1 × 10^4^ particles/μL, thus enabling selective exosome capture. To enhance detection sensitivity, the researchers employ the DNase I enzyme, which selectively degrades free DNA molecules but not those bound to exosomes. This enzyme-mediated signal amplification approach effectively increases the fluorescence signal generated from the captured exosomes, enabling their sensitive detection, as represented in Figure 6.

Despite significant advancements in the development of exosome detection chips, several challenges persist in achieving robust and commercially viable platforms. The optimization of capture efficiency, characterized by the ability to capture a high percentage of exosomes from complex biological samples, remains a critical concern. Non-specific binding poses another hurdle, necessitating the minimization of undesired interactions to ensure specific and accurate exosome detection. Standardizing protocols for sample preparation and analysis represents an ongoing research focus to establish consistent and reproducible workflows across different laboratory settings. Addressing these challenges is imperative to drive the further advancement and widespread adoption of exosome detection chips in diverse biomedical research applications and clinical diagnostics.

### 8.3. Other Cancer-Related Biomarkers Detection

For individuals diagnosed with CRC, the presence and characterization of malignancy-related biomarkers play a pivotal role in understanding the biological activities and pharmacological responses associated with therapeutic interventions. These biomarkers, including free tumor nucleic acids, mRNA expression profiles, proteins, and other substances found in bodily fluids, offer valuable clinical insights to physicians regarding disease status and aid in making informed decisions regarding subsequent treatment strategies [88]. The utilization of microfluidics systems in the measurement of cancer biomarkers has gained significant attention due to their remarkable capabilities in bioanalysis. Microfluidic platforms enable precise detection and analysis of CRC-specific biomarkers, facilitating early diagnosis, treatment monitoring, and personalized therapeutic interventions [79,89].

For instance, efforts have been focused on the monitoring of changes in the mechanobiology of CRC cells using on-chip techniques [90]. The study utilized a microfluidic chip-based platform to analyze mechanical properties and behaviors such as dynamic force deformation, which can serve as a method for detecting mechanical changes linked to the progression of CRC. The research involved subjecting the cells to controlled mechanical forces and monitoring their responses. The researchers were able to identify distinct mechanobiological characteristics associated with different stages of CRC progression. The findings revealed significant changes in the mechanical properties of CRC cells as the disease advanced. The authors observed alterations in cell stiffness, cytoskeletal organization, and migratory behavior, which were correlated with disease stage and aggressiveness. These physical biomarkers hold promise for early detection, prognosis, and monitoring of CRC progression. Another example includes a microfluidic amperometric immunosensor for the determination of claudin7, a biomarker associated with CRC [91]. The immunosensor utilizes porous nanomaterials and was integrated into a microfluidic device, offering the potential for point-of-care applications where it could provide a rapid and reliable diagnosis of CRC in a clinical setting.

The integration of microfluidics technology with CRC represents a significant advancement with substantial potential for enhancing various aspects of CRC research, diagnosis, and treatment. The utilization of microfluidic platforms enables precise manipulation and analysis of biological samples, offering unique advantages such as enhanced sensitivity, high throughput, and improved control over experimental conditions [92]. This technology has demonstrated promising applications in CRC, including the development of advanced diagnostic tools [93]. However, microfluidics in CRC faces certain challenges that need to be addressed for broader adoption and clinical translation. These challenges include standardization of microfluidic protocols, scalability of manufacturing processes, integration with existing clinical workflows, and validation of clinical utility.

## 9. Exploring the Potential of AI in CRC: Advancements, Challenges, and Future Directions

In the field of CRC pathology, the transformative technology of artificial intelligence (AI) has made significant advancements, shaping current trends in diagnosis. The employment of AI algorithms in the analysis and diagnosis of digital pathology images of colorectal tissue samples represents a noteworthy utilization within the scientific domain [94]. These AI algorithms utilize sophisticated machine-learning techniques to identify and classify malignant cells, offering enhanced diagnostic precision and efficiency. By incorporating AI into the diagnostic process, issues of variability in traditional evaluations can be addressed, leading to more dependable and consistent diagnostic assessments and potentially improving patient outcomes [95].

**Figure 7:**
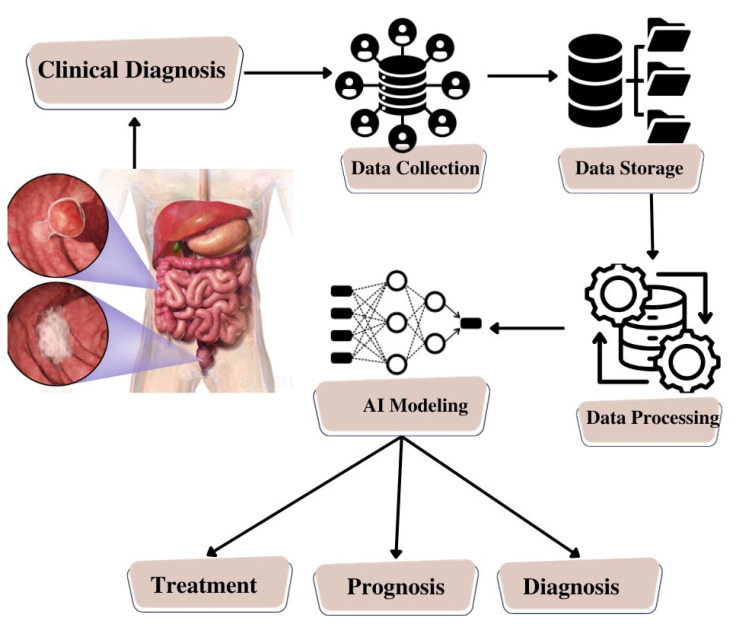
Schematic Illustration of Artificial Intelligence Model Building for Colorectal Cancer.

The integration of microfluidic systems with AI represents a transformative approach to disease diagnosis. The data generated by microfluidic devices, which may include sensor outputs or images, undergoes sophisticated analysis by AI algorithms. These AI algorithms are meticulously trained to process the data, extract relevant features, and make predictions based on patterns and models derived from extensive datasets. What sets AI apart is its capacity for continuous learning and adaptability, leading to heightened diagnostic accuracy and efficiency [75]. The amalgamation of microfluidic systems and AI algorithms holds great promise for enhancing diagnostic capabilities, promising earlier and more precise disease detection, as schematically represented in Figure 7.

In concordance with these advancements, a noteworthy trend in this scientific domain pertains to the development of AI-based prognostic and predictive models. These models synthesize diverse datasets encompassing clinical records, genomic information, and histopathological features, with the objective of generating personalized prognostic predictions pertaining to patient outcomes. Through a comprehensive consideration of multiple factors, including genetic profiles, clinical data, and histopathological findings, these AI-driven models offer invaluable insights into the prognosis of individuals afflicted by CRC. Moreover, they exhibit the potential to forecast individual responses to diverse treatment modalities, thereby facilitating the delivery of personalized therapeutic interventions and enhancing the quality of patient care.

Furthermore, AI has evolved into a valuable tool for the identification of novel biomarker targets in CRC. Leveraging vast genomics and transcriptomics datasets, AI algorithms demonstrate proficiency in unveiling intricate patterns and associations that may elude human researchers due to their complexity and scale [75]. This computational approach streamlines the identification of potential biomarkers crucial for early detection and prognostic assessment in CRC. Additionally, AI’s capabilities extend to the exploration of new drug targets and innovative treatment approaches, thus contributing to the ongoing advancement of CRC management strategies.

As a result, the integration of AI with microfluidics and biosensors in the realm of disease diagnosis particularly unveils a multifaceted paradigm with profound scientific implications, such as:

Enhanced Sensing and Detection: Microfluidic platforms endowed with biosensors have the capacity to provide high sensitivity and specificity in the detection of CRC-associated biomarkers. AI algorithms, in tandem, can optimize the analysis of sensor-derived data by discerning subtle patterns or nuanced variations that may hold diagnostic relevance, especially in the early stages of the disease. This amalgamation, therefore, holds the promise of substantially augmenting the precision of CRC detection, enabling the identification of specific biomarkers within bodily fluids or tissue specimens [96,97].

Real-Time Monitoring: Microfluidic systems, when harmonized with biosensors, empower real-time surveillance of dynamic biological processes. The utilization of AI algorithms facilitates the expeditious processing of the continuous influx of data generated by these sensors. This, in turn, furnishes healthcare practitioners with contemporaneous insights into the progression of CRC, the efficacy of therapeutic interventions, or the emergence of potential complications. Such real-time feedback has the potential to be invaluable in facilitating prompt clinical decision-making for individuals afflicted by CRC [98,99].

Point-of-Care Diagnostics: The confluence of microfluidics, biosensors, and AI holds great potential for the creation of point-of-care diagnostic instruments for CRC. These compact, user-friendly devices can swiftly ascertain CRC-specific biomarkers, thereby enabling early diagnosis even in resource-constrained locales or remote geographical areas [100]. AI algorithms play an integral role in the interpretation of sensor-generated data, furnishing rapid diagnostic outcomes to end-users.

Personalized Treatment Strategies: AI-driven models possess the capacity to scrutinize data derived from microfluidic sensors, thereby affording the ability to tailor therapeutic regimens to the unique attributes of individual CRC patients. By considering factors such as genetic profiles, treatment response data, and trends in biomarker levels, AI algorithms can proffer personalized therapeutic recommendations, optimizing the likelihood of favorable treatment outcomes while minimizing the occurrence of adverse side effects [101].

Early Warning Systems: The continuous monitoring capabilities conferred by microfluidic systems and biosensors, when harmonized with AI, have the potential to serve as early warning systems for CRC recurrence or metastasis. AI algorithms can discern subtle alterations in biomarker levels or patterns of disease progression, thereby facilitating timely interventions and subsequently enhancing the long-term prospects for individuals grappling with CRC [102].

Numerous studies have demonstrated the effectiveness of AI in identifying novel biomarkers in CRC [103]. For instance, a multiple diagnosis model for CRC using an artificial neural network has been established [104]. A comprehensive dataset comprising clinical records, histopathological features, and genomic profiles of CRC patients was collected and preprocessed. The dataset was divided into training, validation, and testing subsets. Multiple ANNs with different architectures, such as feed-forward neural networks, convolutional neural networks, and recurrent neural networks, were constructed and trained using the dataset. The study explores the application of AI in endoscopy and laparoscopy, aiming to develop advanced tools that can assist clinicians in identifying and characterizing CRC lesions.

Others explored the application of AI in the intra-operative tissue classification of CRC using indocyanine green (ICG) perfusion [105]. The study focused on utilizing ICG, a fluorescent dye, to assess tissue perfusion during colorectal cancer surgery. By analyzing real-time ICG perfusion images, AI algorithms are trained to distinguish between cancerous and non-cancerous tissues; this showcases the potential of AI algorithms to assist surgeons in real-time tissue classification, enabling them to make informed decisions during surgery.

AI algorithms can also be used to effectively analyze histopathology images and provide reliable CRC diagnoses. The AI models can learn to identify specific features and patterns indicative of CRC, enabling them to distinguish between cancerous and non-cancerous tissues. The study highlights the potential of AI in enhancing CRC diagnosis by providing reliable and objective analysis of histopathology images. By reducing subjectivity and improving efficiency [106].

AI also has the potential to accurately assess the tumor-stroma ratio (TSR) and its association with survival outcomes in CRC [107]. The research utilizes deep learning techniques to train AI models on a large dataset of CRC histopathology images. The AI models learn to identify tumor and stromal regions within the images, enabling them to accurately calculate the TSR. The association between AI-based TSR quantification and survival outcomes in CRC patients can help determine its prognostic value, which indicates that AI can be used as a tool to objectively quantify the TSR and its correlation with survival in CRC.

As an outcome, the investigation of AI in the realm of CRC presents substantial opportunities for advancing diagnostic accuracy, prognostic assessment, and individualized treatment approaches. However, several challenges need to be addressed, including data quality, interpretability, and regulatory considerations. Despite these challenges, with continued research and advancements, artificial intelligence has a promising future in revolutionizing CRC patient care, ultimately leading to improved patient outcomes.

## 10. Overcoming Hurdles in the Integration of Novel CRC Diagnostic Methods

The translation of innovative methods and devices for the diagnosis of CRC into clinical practice is frequently impeded by various formidable challenges and barriers. Despite the continuous evolution of medical research and technology, several factors hinder the rapid assimilation of these innovations into routine clinical care. The salient barriers encompass:

1. Regulatory Approval and Validation: Novel diagnostic modalities and devices must successfully navigate a labyrinthine process of exhaustive validation and regulatory approval, predominantly orchestrated by entities. This arduous journey is indispensable to ascertaining their safety and efficacy. Securing regulatory clearance or approval is a protracted and resource-intensive endeavor [108].
2. Clinical Evidence and Research: The cornerstone of integration into clinical practice resides in the establishment of robust clinical evidence that substantiates the efficacy and advantages of emerging methods and devices. Executing large-scale clinical trials and research studies to amass voluminous data is an endeavor that is both resource-intensive and time-consuming. Clinicians are predisposed to demand a substantial body of evidence before considering the assimilation of novel technologies to assure enhanced patient outcomes [109].
3. Cost and Accessibility: The fiscal implications of deploying innovative methodologies and apparatuses pose a significant impediment. Particularly when these necessitate specialized equipment or training, the fiscal burden can be formidable. Furthermore, disparities in accessibility to these technologies in distinct healthcare settings or regions can exert a detrimental influence on their integration [110].
4. Integration with Existing Systems: The incorporation of nascent technologies into established healthcare systems, electronic health records (EHRs), and clinical workflows is fraught with challenges. Compatibility issues and the necessity for seamless integration can ensnare the process, impeding the adoption of these innovations [3].
5. Resistance to Change: Entrenched practices and routines within healthcare systems often render clinicians resistant to change. The task of persuading healthcare providers to embrace novel methods can be a gradual and demanding process, contingent on demonstrating unequivocal benefits surpassing those offered by existing approaches.
6. Ethical and Legal Considerations: Ethical and legal quandaries may loom large when deploying new technologies, particularly pertaining to issues of patient privacy, informed consent, and liability. Addressing these concerns effectively is imperative to facilitate adoption [111].
7. Patient Acceptance: The acceptance and comfort of patients with emerging diagnostic methodologies and devices wield significant influence. Patient apprehension or discomfort with these innovations can impede their adoption.
8. Long-Term Follow-Up: Long-term surveillance is of paramount importance, especially in the context of cancer diagnostics such as CRC. Vigilant, extended follow-up is imperative to evaluate the accuracy and efficacy of novel methods, thus prolonging the timeline required for widespread adoption [112].

Mitigating these formidable barriers necessitates a concerted, interdisciplinary effort involving collaboration among researchers, healthcare providers, regulatory authorities, and industry stakeholders. Sustained initiatives for education and awareness are vital to accentuate the advantages of novel diagnostic modalities in enhancing patient care and outcomes. As research continues to advance and the maturation of technologies ensues, these barriers may progressively diminish, facilitating the expanded adoption of innovative CRC diagnostic methods within the realm of clinical practice.

## 11. Challenges and Future Prospects

**Figure 8:**
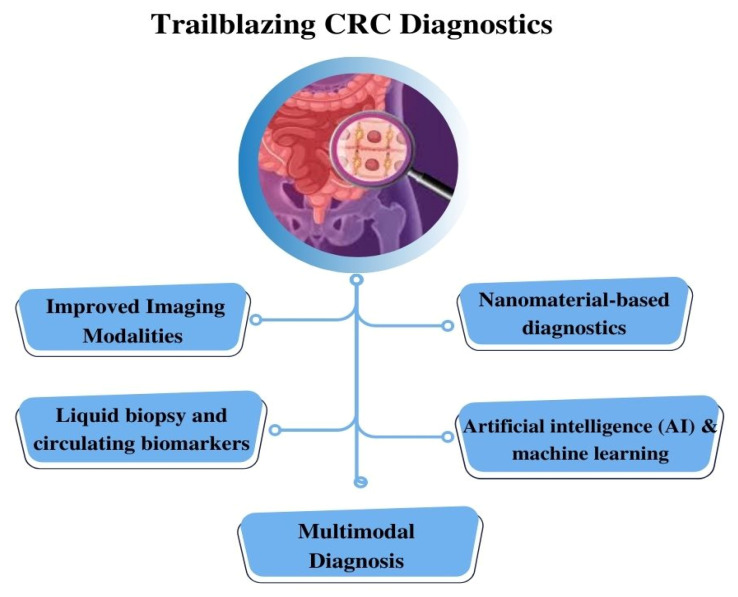
Schematic Illustration of Unleashing the Potential of CRC Diagnostics: Pioneering Strategies for Diagnosis Interventions.

Recent advancements in diagnostic methodologies have led to the development of novel systems tailored specifically for CRC diagnostics, as represented in Figure 8. These innovative platforms hold great promise in addressing critical concerns regarding sensitivity and specificity, thereby revolutionizing the field of cancer diagnostics and leading to improved patient outcomes and enhanced global health. However, the development of microfluidic systems for CRC diagnostics is accompanied by various challenges that need to be overcome. These challenges are associated with the advancement of microfluidic system technologies to provide more accurate, efficient, and patient-centric approaches, ultimately resulting in improved clinical outcomes.

One of the primary challenges in the development of microfluidic systems for CRC diagnostics is efficiently handling samples within these devices, including manipulation, transport, and containment of small volumes while preserving their integrity. Proper mixing, minimal losses, and prevention of cross-contamination are critical considerations. Another challenge lies in detecting specific CRC biomarkers, such as CTCs, ctDNA, and exosomes, with high sensitivity and specificity within microfluidic systems. Selecting appropriate capture agents, optimizing capture surfaces, and reducing non-specific binding are crucial for accurate biomarker detection. Additionally, enabling multiplexed analysis for simultaneous detection of multiple biomarkers is desirable. Thus, addressing these challenges will advance microfluidic systems for CRC diagnostics, providing accurate, efficient, and patient-centric tools for improved clinical outcomes and personalized treatment strategies.

The future prospects of microfluidic systems in CRC diagnostics appear highly promising. Continued advancements in sample handling techniques, biomarker detection methods, and multiplexed analysis capabilities are expected to revolutionize the field. Through ongoing research and development endeavors, microfluidic systems are poised to become increasingly efficient, accurate, and user-friendly. These systems will enable early detection of CRC, facilitate personalized treatment strategies, and significantly enhance patient outcomes. Moreover, the integration of microfluidic technology with complementary diagnostic modalities, such as genomics and imaging, holds great potential for comprehensive CRC understanding and management.

## 12. Conclusions and Viewpoints

**Figure 9:**
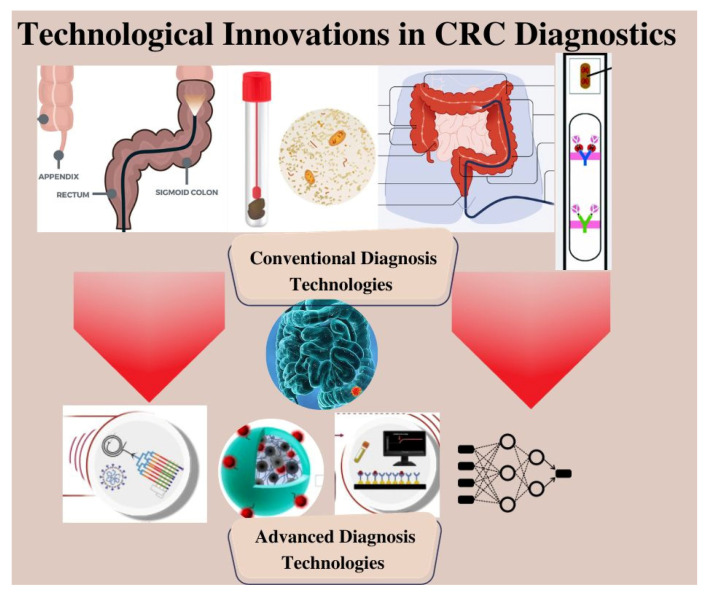
Schematic Representation of Next-Generation CRC Diagnosis.

This comprehensive review article conducts an in-depth analysis of the conventional methodologies currently employed in the diagnosis of CRC. The article highlights the limitations of these established approaches, emphasizing the need for innovative and more effective strategies. It further explores the emerging fields of microfluidics and AI as promising avenues for advancing CRC research and improving patient care, as represented in Figure 9.

Through the utilization of cutting-edge microfluidic technologies, researchers can create novel diagnostic methods that offer improved sensitivity and precision. The incorporation of microfluidics with biosensors, molecular assays, and imaging techniques allows for the identification of specific biomarkers and the exploration of tumor heterogeneity and drug responses in CRC. Furthermore, the application of AI in CRC brings advanced computational algorithms to analyze vast amounts of clinical and molecular data. AI-powered models are helping in identifying patterns, predicting outcomes, and developing personalized treatment strategies for CRC patients. However, several challenges are present that need to be addressed for the successful translation of microfluidics and AI into clinical practice i.e., standardization, validation, scalability, and regulatory approval are key areas that require attention to ensure the reliability and widespread implementation of these technologies in CRC research and patient care.

In summary, the integration of microfluidics and AI holds immense promise for advancing CRC research and improving patient outcomes. By overcoming the challenges associated with these technologies, one can fully leverage their potential and transform CRC diagnosis, treatment, and personalized medicine, ultimately reducing the burden of this disease.

## Figures and Tables

**Table 1:** Tabulated Summary of Conventional Technologies for Colorectal Cancer: Strengths and Limitations.

Method	Cost	Sensitivity	Specificity	Advantage	Limitation	Ref.
Guaiac-based fecal occult blood test	Low	65–100%	90.12–97%	Simple, inexpensive, non-invasive, and widely available screening for colorectal cancer.	False positives and false negatives can occur, requiring further confirmatory testing.	[33]
Fecal immunochemical	Low	74–88%	93–96%	Highly specific, sensitive, convenient, and non-invasive screening for colorectal cancer.	Limited sensitivity for detecting precancerous lesions.	[34]
Multi-target stool DNA test	High	70–92%	82–97%	Non-invasive detection of multiple genetic markers for colorectal cancer.	Higher cost compared to other screening methods for colorectal cancer.	[35]
Colonoscopy	High	95%	80–100%	Direct visualization of the colon for accurate detection of abnormalities.	Potential risks such as bowel perforation, bleeding, and sedation-related complications.	[36]

## Data Availability

Not applicable.
